# Are women adequately informed before gynaecological surgery?

**DOI:** 10.1186/s12905-017-0426-7

**Published:** 2017-08-25

**Authors:** Mojgan Pakbaz, Ewa Rolfsman, Mats Löfgren

**Affiliations:** 1 Department of Clinical Sciences, Obstetrics and Gynaecology, Umeå University, SE-901 87 Umeå, Sweden; 2 Department of Applied Educational Science, Umeå University, Umeå, Sweden

**Keywords:** Preoperative information, Benign gynaecological surgery

## Abstract

**Background:**

Surgery for pelvic organ prolapse, urinary incontinence, and hysterectomy are the most common gynaecological surgeries that can affect the function of the bladder and bowel as well as one’s sexual life. There is evidence that adequate patient information given preoperatively regarding expected outcomes of surgery is important because well-informed patients are more satisfied with the results of surgery and recover faster. However, there is little known about the amount and quality of information given to women before surgery. This study investigates whether women received information before gynaecological surgery on the effect of surgery with respect to the functioning of the bladder (micturition, ability to stay continent) and the bowel (empty bowel) as well as the surgery’s effect on sexual functioning.

**Methods:**

A prospective, cross-sectional study was conducted. Women undergoing hysterectomy, surgery for vaginal prolapse, or surgery for urinary incontinence (*n* = 972) and included in the Swedish National Register for Gynaecological Surgery participated in the study. A questionnaire was developed and distributed to the women along with the preoperative questionnaire from the register.

**Results:**

About 50% of the women undergoing prolapse surgery were supplied with information regarding the effect of the surgery with respect to remaining continent, to emptying bowels, micturitaion, and sexual life. One out of four women undergoing hysterectomy received information about the effect of the surgery on the sexual life and bladder function. In the incontinence group, the given information about the surgery’s effect on bladder function and sexual function was 80 and 30%, respectively.

**Conclusion:**

Surgery in the vagina and the genital organs may affect function of the organs close to the surgical area (i.e., bladder and bowel) and may affect sexual function. According to this study, women are inadequately informed before surgery. Access to information via oral and written counselling needs to be improved.

**Electronic supplementary material:**

The online version of this article (10.1186/s12905-017-0426-7) contains supplementary material, which is available to authorized users.

## Background

Each year, approximately 17,000 women in Sweden undergo surgery for pelvic organ prolapse (POP), urinary incontinence (UI), or hysterectomy on benign indication [1].

Symptoms associated with POP are complex. Women with prolapse may present with a variety of symptoms from the bladder (frequency, urgency, and urinary incontinence) and/or the bowel (problems emptying and faecal incontinence) [2]. Prolapse may have a negative effect on a woman’s sexual life [2–5]. It is also known that after surgery while some symptoms improve, others may remain unchanged. Furthermore, there is also a risk that new undesirable symptoms may arise [2]. Concerning hysterectomy, its effect on sexual activity and occurrence of urinary incontinence has been debated. Some studies have found that hysterectomy has no effect on sexual activity and symptoms of UI, whereas a negative effect on sexuality and appearance of UI has been reported in other studies [6–8]. Surgery for UI has been shown to have a positive effect on sexual function [9–11], but its effect on bowel function is unknown.

Previous studies have suggested that women, particularly those with vaginal prolapse, have little access to publicly available information on prolapse [3, 12]. Informing women not only about the expected positive effects but also about conceivable side effects of an operation is an essential part of the decision-making that may lead to surgery [13, 14], since there is evidence that well-informed patients are more satisfied with the results of surgery and recover faster [13]. This study investigates the quality and amount of information that women receive from the surgeon before a gynaecological surgery on benign indication regarding the effect of the operation on urinary function, bowel function, and sexual function.

## Methods

This was a prospective, cross-sectional study based on a combination of medical data included in the Swedish National Register for Gynaecological Surgery (the Gynop-register) and data from a complementary questionnaire distributed to women undergoing benign surgery [15]. The study included women with planned hysterectomy (*n* = 385), surgery for POP (*n* = 307), or surgery for UI (*n* = 280). The complementary questionnaire was validated and distributed to the women along with the preoperative questionnaire. The complementary questionnaire consisted of open-ended and multiple-choice questions (Additional files 1 and 2). The questionnaire included items requesting the women’s own description of POP and its causes, and the source of information on their own condition for which they contacted the gynaecology department [12]. In addition, the questionnaire included questions about whether they have been informed by the physician about the surgery and its effect on sexuality, urinary function (ability of being/staying continent and micturition), and bowel function (emptying bowel) with a “yes” or “no” answer. The study period was March 1 through November 31, 2010 and the response rate was 95%. The data set included medical data reported by patients and surgeons, patient-reported symptoms, and questions regarding whether women had an active role in the decision-making process before surgery.

Women in the prolapse group underwent surgery in one or several compartments and of these 14% underwent vaginal hysterectomy. All women in the incontinence group had surgery with synthetic sling (retropubic or transobturator) without concomitant prolapse surgery. Of the women in the hysterectomy group, 71% underwent abdominal surgery, 2% underwent laparoscopic hysterectomy, and 27% underwent vaginal hysterectomy. Data were analysed using SPSS v. 19.0 and are presented as mean ± standard deviation or number and percentage.

## Results

Characteristics of the participants are given in Table 1. In the prolapse group, the proportion of women who received information regarding the effect of surgery on bladder, bowel, and sexual function ranged from 38 to 53% (Table 2). Almost 50% of the women in the prolapse group reported symptoms of UI, difficulty emptying bowel, and/or micturition problems, but only half of them had received information about the effect of surgery on these symptoms (Table 3). One out of six women in the prolapse group reported that prolapse had negatively affected their sexual life, but almost 40% of them had been informed about whether surgery may affect their sexual life (Table 3).

**Table 1:** Characteristics of the participants

Characteristic	Prolapse (*n* = 307)	Incontinence (*n* = 280)	Hysterectomy (*n* = 385)
Age	59 ± 10.2	50 ± 10.3	46 ± 7.2
BMI	26 ± 3.8	27.4 ± 10.2	27 ± 4.9
Education			
9 years or less	88 (29)	46 (16)	52 (14)
High school degree	81 (26)	106 (38)	169 (44)
University degree	124 (40)	126 (45)	156 (41)
Menopause	239 (78)	128 (46)	11 (3)
Current smoker	21 (6)	14 (5)	70 (18)
Co-morbidity			
Diabetes mellitus	10 (3)	10 (4)	6 (2)
Hypertension	85 (28)	65 (23)	66 (17)
Previous caesarean section	19 (6)	27 (10)	44 (11)

All values are expressed as mean ± standard deviation or as number (%)

**Table 2:** Information given regarding the effects of surgery on micturition, sexuality, ability to stay continent, and emptying bowel

	Prolapse (*n* = 307)	UI^a^ (*n* = 280)	Hysterectomy (*n* = 385)
Micturition	152 (50)	176 (63)	89 (23)
Sexuality	123 (40)	83 (30)	98 (25)
Ability to stay continent	163 (53)	230 (82)	88 (23)
Emptying bowel	118 (38)	56 (20)	65 (17)

All values are expressed as number (%)

^a^ *UI* urinary incontinence

**Table 3:** Information given in relation to the symptoms reported by the study population

	Prolapse (*n* = 307)	UI (*n* = 280)	Hysterectomy (*n* = 385)
Reported UI	134 (44)	268 (96)	104 (27)
Received information	77 (57)	222 (83)	20 (19)
Reported micturation problem	128 (42)	41 (15)	73 (19)
Received information	70 (55)	25 (61)	21 (29)
Reported difficulty emptying bowel	139 (45)	75 (27)	113 (29)
Received information	60 (43)	13 (17)	16 (14)
Reported negative impact on sexuality	50 (16)	20 (7)	-----^a^
Received information	19 (38)	6 (30)	-----

All values are expressed as number (%)

^a^The Ethical Board did not approve this question for inclusion in the register for women undergoing hysterectomy for benign indication (fibromas and dysfunctional bleeding)

The majority of women in the incontinence group reported that they had received information concerning the effect of incontinence surgery on their ability to stay continent (82%) and micturition problems (63%) (Table 2). However, the information given about the effect of surgery on bowel function or sexuality in this group was only in 20 and 30% of cases. UI negatively affected sexual life in 7% of women in the incontinence group; however, only 30% of these women had received information about the possible effects of surgery on their problem (Table 3).

A relatively small proportion of the women in the hysterectomy group reported that they had been informed by the surgeon about the effects of the operation on urinary, bowel, and sexual function (17–25%) (Table 2). In this group, 29% reported bowel symptoms (feeling of incomplete emptying), 27% reported UI, and 19% reported micturation difficulties (Table 3). However, the information given by the surgeons about the effect of surgery for each of these problems had reached only 14, 19, and 29% of the patients, respectively. The majority of women (95%) in each group reported that they had been involved in the decision to have surgery.

## Discussion

In this study, we investigated whether women received information from the surgeon before surgery for prolapse, urinary incontinence, or hysterectomy concerning the possible effects of surgery on bladder function, bowel function, and sexual function. We chose these three groups of women because these surgeries are common and they may also influence urinary tract function, bowel function, and sexual function either in a positive or a negative way. The only symptom related to vaginal prolapse that one can be sure of being relieved of after surgery is the sensation of bulging. Other symptoms related to bowel, bladder, or sexual function among women with prolapse can either be improved [11, 16–18], remain unaltered, or even become worse after surgery [19, 20]. Against this background, every woman who undergoes surgery for prolapse should be adequately informed about the effects and side effects of the surgery regarding sexual function and functioning of the bladder and the bowel. In this study, the proportion of women who did receive information from the surgeon regarding the probable effect of the surgery on the functioning of the bowel, the bladder, and sexual life is low. This proportion ranged in the interval of one out of three to every other woman.

It has been debated whether hysterectomy and vaginal hysterectomy in particular (due to prolapse or for any other reason) contributes to the development of urinary incontinence [21]. The effect of hysterectomy on sexual function has also been discussed. According to some studies, hysterectomy may influence sexuality in a negative way [6]. However, other studies have indicated that sexual function remains unaltered after hysterectomy [16]. It is also known that bowel function may be disturbed for a short while after abdominal hysterectomy, but pre-existing bowel dysfunction is often related to other causes and a hysterectomy does not alter this dysfunction [22, 23]. Nevertheless, few women undergoing hysterectomy in this study received preoperative information on surgery’s influence on bladder, bowel, and sexual function.

Furthermore, there is a major difference between hysterectomy group and POP group regarding the amount of information they are given preoperatively and this may be explained by different symptoms in each group leading to the decision for surgery. Fibroma and bleeding disorders were the main reasons for surgery in the hysterectomy group, whereas in the prolapse group in addition to the bulging symptom other symptoms from bladder and bowel can interfere with the decision for surgery. Therefore, the prolapse group received information in a higher degree regarding effect of surgery on the function of the bowel, bladder, and sexual life than women undergoing hysterectomy.

Midurethral sling is a surgical method for treatment of stress-related urinary incontinence. Women with coexisting urge incontinence have a reduced success rate after surgery [24]. In our study, women in the incontinence group were provided with information regarding the effects of surgery on their UI to a high degree. The effect of midurethral sling surgery on sexuality is debated [25, 26]. Improvement of sexual life due to cure of coital incontinence after surgery has been reported [25]. One of the rare but known complications after sling plastic, in particular the transobturator sling, is dyspareunia [26]. In this study, women were seldom provided with information regarding influence of surgery on sexual function.

### Strengths and limitations

Data used in this study are from the Gynop-register, which is a national register and includes all women undergoing gynaecological surgery in Sweden at both university hospitals as well as county hospitals. Therefore, there is no selection bias for patients included in this study. A limitation of this study is the recall bias. It could be that the surgeon does mention some information on the effect of surgery on bladder, bowel, and sexual function, but the patients do not remember because the amount of information can be overwhelming.

## Conclusion

The present study shows that women are not adequately informed before gynaecological surgery. Given the conflicting evidence on the effect of the surgeries, it is important to convey these uncertainties to the patient. There is need to improve the access of information to women through oral counselling and/or written information before surgery. Therefore, national patient information on postoperative course and effect of prolapse surgery on nearby organs has been conducted and is now more accessible for patients undergoing POP surgery in Sweden. National patient information for other gynaecological surgery is now being developed. Further prospective studies are needed that investigate whether this effort may lead to satisfaction in outcome of surgery.

## Additional files

Additional file 1: The study questionnaire translated in English. It consists of open-ended and multiple-choice questions responded by three groups of patients with a scheduled surgery for vaginal prolapse, urinary incontinence or bleeding disorder with a hysterectomy. (DOC 47 kb)

Additional file 2: The study questionnaire in Swedish language. (DOC 54 kb)

## Abbreviations

POP: Pelvic organ prolapse
The Gynop-register: The Swedish National Register for Gynaecological Surgery
UI: Urinary incontinence
