# Supplementary material for: Are women adequately informed before gynaecological surgery?
Source: BMC Womens Health. 2017 Aug 25;17:68. doi: 10.1186/s12905-017-0426-7 (PMC6446650; doi:10.1186/s12905-017-0426-7)
Supplement: Supplementary file 1 — The study questionnaire translated in English. It consists of open-ended and multiple-choice questions responded by three groups of patients with a scheduled surgery for vaginal prolapse, urinary incontinence or bleeding disorder with a hysterectomy. (DOC 47 kb) [file 12905_2017_426_MOESM1_ESM.doc]

**Additional file 1**

**The heading and question six were changed for the three groups of patients depending on the surgical procedure for which the patient is scheduled.**

**Hospital:……………………… Patient name:…………………………….**

**Research study: Your path from symptoms to planned incontinence/ prolapse / uterine surgery.**

**We would like to ask you some questions about your upcoming surgery, how you got there and the information you have received. The questions are part of a study that has the overall aim of improving the information that you receive as a patient prior to surgery. The study is separate from the quality register and the information will not be entered into your patient chart.**

**When you have answered the questionnaire, return it together with the larger questionnaire (your health statement). The answers will be processed anonymously.**

**1: Select the highest level of education that you have completed.**

🞏 Nine-year compulsory school (or primary school, secondary school)
🞏 Folk High School (Folkhögskola)
🞏 Upper Secondary School
🞏 University or College

**2a):** Have you had the symptoms that caused you to seek medical care for more than one year?🞏 Yes 🞏 No

If not, go to the next page, question 3

**2a) Number of year…..**

**2b) why didn’t you seek medical attention sooner?** *Select one or more responses*

🞏 My problems had to be put on the backburner while the needs of other family members took priority

🞏 Felt that the financial loss associated with sick leave was too large

🞏 Presented for this problem earlier but the treatment provided was inadequate

🞏 Did not feel the symptoms were so serious

🞏 Didn’t feel I was able to take time off from work

🞏 Sought care previously and advised to wait and see

🞏 Gynaecological examination experienced as unpleasant

🞏 Did not know that the symptoms could be treated

🞏 *Did not know that the problem was prolapse.* *(this question only for patients scheduled for prolapse surgery)*

🞏 Embarrassing problem to discuss

🞏 Had planned to have more children

🞏 Other:……………………………………………………..

**2c)** **why are you seeking care now?** *Select one or more responses*

🞏 Tried other treatment, but the results were inadequate

🞏 Was offered surgery in the past but chose to wait.

🞏 Increased impact on my social life **(**friends and family, leisure, sport etc**).**

🞏 Increased impact on my working life (ability to do my job).

🞏 Encouraged by someone to seek care.

🞏 Increased impact on my sex life and relationship.

🞏 Actually presented for a different reason.

🞏 Worsening symptoms
🞏 Other:……………………………………………………..

**3: Did you suspect that your symptoms and problems were due to prolapse before the doctor made the diagnosis of prolapse**?🞏 **Yes 🞏 No**

**If not, what did you think was the cause of your problem?** *Select one or more responses*

🞏 Urinary tract infection/ urinary tract symptoms

🞏 Due to heavy lifting or heavy work

🞏 Previous pregnancies

🞏 Age-related changes
🞏 Constipation

🞏 Tumour
🞏 Other ………………………

**4a:** **Has a decision been taken to operate?** 🞏 Yes 🞏 No
**If not,** go to the next page, question 5

**4b: Do you believe that the surgery may affect your ability to:**

Have a bowel movement? 🞏 Yes 🞏 No 🞏 Don’t know

Prevent urinary leakage (incontinence)? 🞏 Yes 🞏 No 🞏 Don’t know

Empty your bladder? 🞏 Yes 🞏 No 🞏 Don’t know

Have sexual intercourse? 🞏 Yes 🞏 No 🞏 Don’t know

**4c: Do you feel that you were involved in the decision to have surgery?** 🞏 Yes 🞏 No

**If not,** would you have liked to have participated in the decision? 🞏 Yes 🞏 No

**4d: Have you and your doctor discussed the effects of the surgical procedure on your symptoms?** 🞏 Yes 🞏 No

**If not,** would you have liked to have had such a discussion? 🞏 Yes 🞏 No

**4e: Has a doctor informed you about the possible effect of the surgical procedure on:**

Having a bowel movement? 🞏 Yes 🞏 No

**If not,** would you have liked to have had such information? 🞏 Yes 🞏 No

Preventing urinary leakage (incontinence)? 🞏 Yes 🞏 No
**If not,** would you have liked to have had such information? 🞏 Yes 🞏 No

Emptying your bladder? 🞏 Yes 🞏 No
**If not,** would you have liked to have had such information? 🞏 Yes 🞏 No

Having sexual intercourse? 🞏 Yes 🞏 No
**If not,** would you have liked to have had such information? 🞏 Yes 🞏 No

**5: Were you given the opportunity to ask the questions you wanted to ask during your visit to the doctor?**

🞏 Yes 🞏 No

**6: Have you received any information over the past year about**
 **prolapse / urinary incontinence / menstruation/bleeding disorder through?**

Relatives, friends or acquaintances? 🞏 Yes 🞏 No

Health care visit? 🞏 Yes 🞏 No

Information brochures? 🞏 Yes 🞏 No

Newspapers / popular press? 🞏 Yes 🞏 No

Weekly publication / monthly magazine? 🞏 Yes 🞏 No

Books? 🞏 Yes 🞏 No

Radio / TV? 🞏 Yes 🞏 No

Internet? 🞏 Yes 🞏 No

Other source of information? 🞏 Yes 🞏 No

If yes, please specify: ……………………………..

**7**. Many women experience prolapse, but it is not always easy for women to realise that the problem is due to prolapse.
.

a) **Describe what you think prolapse is!**  **…………………………………………**

**b) What do you think causes prolapse? …………………………………………..**

**Date of completion of the questionnaire: …………………………..**

**Thank you for your help!**

**If you have any questions regarding this study please contact one of us.**

**Senior physician Mats Löfgren, mats.lofgren@vll.se**

**Assistant senior physician Mojgan Pakbaz, mojgan.pakbaz@vll.se**

**Department of Obstetrics and Gynaecology**

**Norrland University Hospital**

**+46 (0)90 7850000**
