# Supplementary material for: Are women adequately informed before gynaecological surgery?
Source: BMC Womens Health. 2017 Aug 25;17:68. doi: 10.1186/s12905-017-0426-7 (PMC6446650; doi:10.1186/s12905-017-0426-7)
Supplement: Supplementary file 2 — The study questionnaire in Swedish language. (DOC 54 kb) [file 12905_2017_426_MOESM2_ESM.doc]

**Additional file 2**

**Rubriken och fråga sex ändrades mellan de tre grupper av patienter beroende på vilken operation patienten är inplanerad för.**

**Sjukhus :……………………… Patientens namn:…………………………….**

**Forskningsstudie: Din väg från besvär till beslutad inkontinens/ framfalls / livmoder operation.**

**Vi önskar ställa några frågor som handlar om din kommande operation, vägen dit och den information du har fått. Frågorna ingår som en del i en studie som övergripande syftar till att förbättra informationen till dig som patient inför en operation. Studien är skild från kvalitetsregistret och informationen kommer inte att läggas i din journal.**

**När du har besvarat enkäten, returnerar du den tillsammans med den större enkäten, d.v.s. din hälsodeklaration. Svaren kommer att bearbetas anonymt.**

**1: Markera de utbildningar som du har avslutat.**

🞏 Grundskola (eller folkskola, realskola
🞏 Folkhögskoleutbildning
🞏 Gymnasieutbildning
🞏 Högskole- eller universitetsutbildning

**2a):** **Har du haft symtomen du sökt för i mer än ett år**? 🞏 Ja 🞏 Nej

Om nej gå till nästa sida, fråga 3

**2a) Antal år**……..

**2b) Varför sökte du inte sjukvård tidigare?** *Markera ett eller flera svarsalternativ*

🞏 Mina besvär har fått stå tillbaka p.g.a. andra familjemedlemmars behov

🞏 Ansåg att den ekonomiska förlusten vid sjukskrivning var för stor

🞏 Har sökt tidigare men behandlingen som gavs är otillräcklig

🞏 Bedömde själv att besvären inte var så allvarliga

🞏 Har inte ansett mig kunna ta ledigt från arbetet

🞏 Har sökt tidigare men fått rådet att avvakta

🞏 Obehagligt att bli gynekologiskt undersökt

🞏 Visste inte att besvären kunde behandlas

🞏 *Visste inte att besvären var framfall* *(denna fråga enbar till planerade för framfallsoperation)*

🞏 Känns genant att prata om problemet

🞏 Hade planerat att föda fler barn

🞏 Annat: ……………………………………………

**2c)** **Vad är anledningen till att du söker nu?** *Markera ett eller flera svarsalternativ*

🞏 Provat annan behandling men den ger inte tillräcklig effekt

🞏 Blivit erbjuden operation tidigare men valde att avvakta

🞏 Ökad påverkan på mitt sociala liv **(**umgänge, fritid, sport etc**)**

🞏 Ökad påverkan på mitt arbetsliv (förmågan att sköta arbetet)

🞏 Blev uppmanad av annan person att söka

🞏 Ökad påverkan på mitt sex- och samliv

🞏 Sökte egentligen av annan orsak

🞏 Tilltagande symtom
🞏 Annat: ……………………………………………………..

**3:Misstänkte du att dina symtom och besvär berodde på framfall innan läkare konstaterade att du hade framfall**? **🞏 Ja 🞏 Nej**

**Om nej, vad trodde du att dina besvär berodde på**? *Markera ett eller flera svarsalternativ*
🞏 Urinvägsinfektion/ urinvägsbesvär

🞏 Effekt av tunga lyft eller tungt arbete

🞏 Tidigare graviditeter

🞏 Åldersförändringar
🞏 Förstoppning

🞏 Tumör
🞏 Annat ………………………

**4a:** **Är det bestämt att du skall opereras?** 🞏 Ja 🞏 Nej
**Om nej**, hoppa till nästa sida, fråga 5

**4b: Tror du att operationen kan påverka din förmåga;**

Att tömma tarmen? 🞏 Ja 🞏 Nej 🞏 Vet ej

Att hålla urinen? 🞏 Ja 🞏 Nej 🞏 Vet ej

Att tömma urinblåsan? 🞏 Ja 🞏 Nej 🞏 Vet ej

Att ha samlag? 🞏 Ja 🞏 Nej 🞏 Vet ej

**4c: Upplever du att du var delaktig i operationsbeslutet?** 🞏 Ja 🞏 Nej

**Om nej**, hade du önskat delaktighet i beslutet? 🞏 Ja 🞏 Nej

**4d: Har du och din läkare diskuterat operationens effekter på dina symtom?**

🞏 Ja 🞏 Nej

**Om nej**, hade du önskat sådan diskussion? 🞏 Ja 🞏 Nej

**4e: Har läkare informerat dig om operationens eventuella effekt på;**

Att tömma tarmen? 🞏 Ja 🞏 Nej

**Om nej**, hade du önskat sådan information? 🞏 Ja 🞏 Nej

Att hålla urinen? 🞏 Ja 🞏 Nej
**Om nej**, hade du önskat sådan information? 🞏 Ja 🞏 Nej

Att tömma urinblåsan? 🞏 Ja 🞏 Nej
**Om nej**, hade du önskat sådan information? 🞏 Ja 🞏 Nej

Att ha samlag? 🞏 Ja 🞏 Nej
**Om nej**, hade du önskat sådan information? 🞏 Ja 🞏 Nej

**5: Fick du under läkarbesöket möjlighet att ställa de frågor du önskade?**

🞏 Ja 🞏 Nej

**6: Har du under det senaste året tagit del av någon information om** **framfall / urininkontinens / menstruations-/blödningsrubbning via:**

Släkt, vänner eller bekanta? 🞏 Ja 🞏 Nej

Besök inom sjukvården? 🞏 Ja 🞏 Nej

Informationsbroschyrer? 🞏 Ja 🞏 Nej

Dags-/ kvällspress? 🞏 Ja 🞏 Nej

Veckopress / månadsmagasin? 🞏 Ja 🞏 Nej

Böcker? 🞏 Ja 🞏 Nej

Radio / TV? 🞏 Ja 🞏 Nej

Internet? 🞏 Ja 🞏 Nej

Annan informationskälla? 🞏 Ja 🞏 Nej

Om ja vilken? : ……………………………..

**7**. Framfall är något som många kvinnor får, men det är inte alltid lätt för kvinnan att själv veta att det rör sig om ett framfall.
.

a) **Beskriv vad du tror att framfall är!**  **…………………………………………..**.

b) **Vad tror du orsakar framfall? …………………………………………..**

**Datum för enkätens besvarande: …………………………..**

**Tack för hjälpen!**

**Om du har frågor avseende denna studie kontakta någon av oss.**

**Överläkare Mats Löfgren, mats.lofgren@vll.se**

**Biträdande överläkare Mojgan Pakbaz, mojgan.pakbaz@vll.se**

**Kvinnokliniken**

**Norrlands Universitetssjukhus**

**090 7850000**
